# Trends in the prevalence and incidence of ulcerative colitis in Japan and the US

**DOI:** 10.1007/s00384-023-04417-6

**Published:** 2023-05-19

**Authors:** Michiyo Yamazaki, Hsingwen Chung, Youran Xu, Hong Qiu

**Affiliations:** Global Epidemiology, Office of the Chief Medical Officer, Janssen Research and Development, LLC, Titusville, NJ USA

**Keywords:** Database, Epidemiology ulcerative colitis, Incidence prevalence, Japan, United States

## Abstract

**Purpose:**

To estimate and compare annual prevalence and incidence, and demographic characteristics of patients with ulcerative colitis (UC) in Japan and the United States (US).

**Methods:**

All patients with UC were identified from large employment-based healthcare claims databases (Japan Medical Data Center [JMDC] in Japan and IBM MarketScan Commercial Claims and Encounters database [CCAE] in the US), from 2010 to 2019. Cases were confirmed using International Classification of Disease-9/10 codes with/without Anatomical Therapeutic Chemical codes. Annual age-standardized prevalence and incidence rates were estimated for the JMDC by direct standardization using the CCAE as the standard population.

**Results:**

Patients with UC were younger in Japan than in the US and men were affected more than women, whereas the reverse was true in the US. Annual prevalence per 100,000 population increased significantly from 5 in 2010 to 98 in 2019 in Japan and from 158 to 233 in the US. Prevalence increased in men more than in women and in all age groups in Japan, whereas increases were observed similarly in men and women, and in the 6 to < 65-year age groups in the US. Annual incidence per 100,000 person-years increased significantly over time in both sexes and in all age groups in Japan, with higher increases in women and in ≥ 18 year-olds. UC incidence rates did not change over time in the US.

**Conclusion:**

Ten-year trends in epidemiology of UC differ between Japan and the US. The data point to a growing disease burden in both countries that warrants investigation of measures for prevention and treatment.

**Supplementary Information:**

The online version contains supplementary material available at 10.1007/s00384-023-04417-6.

## Background

Ulcerative colitis (UC) is a subtype of inflammatory bowel disease (IBD) characterized by inflammation and ulceration of the mucosa and sub-mucosa of the rectum, extending to the colon [1]. The clinical presentation is typically bloody diarrhea, with or without other symptoms of gastrointestinal disturbance, systemic symptoms of inflammation, and extra-intestinal manifestations such as arthritis or sclerosing cholangitis [1]. The extent of mucosal inflammation tends to wax and wane and is a key factor in determining severity and the disease course. The peak age of onset is between 15 and 30 years with a second peak between 50 and 70 years, and with men and women equally affected [2]. Risk factors include a history of smoking, a family history of IBD, the presence of certain genetic polymorphisms, and exposure to some drugs such as oral contraceptives and non-steroidal anti-inflammatories [1].

The prevalence of UC shows marked regional variation, being higher in Northern Europe and North America (> 198 per 100,000 population) than in Asia (20–101 per 100,000 population) [3]. Incidence rates of UC are increasing globally, particularly in regions undergoing rapid industrialization such as Asia, Africa, and South America [3–6].

In the United States (US), the prevalence and incidence of UC have increased steadily since the 1970s [7]. UC prevalence between 2007 and 2016 increased by 152% in children aged 2–17 years, from 8.6 to 21.6 per 100,000 population, with the highest increase in the 10–17-year age group. Over the same period, the prevalence of UC in adults (≥ 18 years) increased by 142%, from 74.8 to 181.1 per 100,000 population [8].

A review of population-based data from Asian countries reported incidence proportions of UC ranging from 0.49 per 100,000 population (Southeast Asia, 2011–2013) to 12.2 per 100,000 population (Japan, 2014) [9]. The incidence of UC in Japan has been steadily increasing since the 1960s and the estimated prevalence was 133.2 per 100,000 population in 2014, a level close to that reported in the US and other Western countries [3, 10]. In 2014, there were 180,000 persons with UC in Japan with 15,000 new diagnoses per year, suggesting that in 2022 the total number of individuals with UC could have risen to 300,000 [10].

It is not clear if the characteristics of patients with UC differ between different countries, although there is evidence of geographic variability in polymorphisms that influence the risk of developing IBD [11]. We conducted a retrospective cohort study to estimate the annual prevalence, incidence, and demographic characteristics of patients with UC over a 10-year period from 2010 to 2019. The study was conducted using employment-based healthcare claims databases in the US and Japan to investigate potential differences in the populations of patients with UC in each country.

## Methods

### Study design and data sources

The Japan Medical Data Center (JMDC) is a payer-based database collecting claims, membership status of the insured individuals, and health check-up results since 2005 from more than 250 payers, covering non-government employees of large corporations aged 18 to 65 years and their dependents (children < 18 years of age and adults aged < 75 years). The dataset cumulatively included approximately 14 million lives in Japan (~ 10% of total population) in February 2022 [12]. Claims data are derived from monthly claims issued by clinics, hospitals, and pharmacies. The JMDC database captures all inpatient, outpatient, and pharmaceutical services provided to its members, including diagnoses, surgical procedures, and medications [13]. Diagnoses are coded in International Classification of Disease 10 (ICD-10) format and medications in Anatomical Therapeutic Chemical format. The data from an insured employee or their dependent can be followed longitudinally even if they transfer hospitals or visit multiple facilities [14]. Data collection ceases if the employment status of members changes and they leave the insurance plan.

The IBM MarketScan Commercial Claims and Encounters (CCAE; currently known as Merative™ MarketScan^®^) database includes health insurance claims across the continuum of care (inpatient, outpatient, outpatient pharmacy, behavioral healthcare) as well as enrollment data of employees of large corporations and health plans across the US who provide private healthcare coverage for more than 155 million lives (~ 46% of total population) up to 65 years of age [15]. Diagnoses are coded in ICD-9 and ICD-10 formats.

The JMDC includes individuals up to 75 years of age; however, our analysis was limited to individuals aged < 65 years because not all healthcare claims from the population 65 years and older in the CCAE were available due to their Medicare Supplemental Insurance coverage. All data were de-identified and fully compliant with relevant patient confidentiality requirements, including the Health Insurance Portability and Accountability Act of 1996 in the US and the Japan Act of the Personal Information Protection. Individual informed consent and ethical approval were not required.

### Study population and case identification

Any individual in the claims databases < 65 years of age was eligible for the study if they had been continuously enrolled in the health insurance plan for at least 12 months at any given time during the study period. Patients with UC were identified using a diagnostic code with or without a relevant pharmacy code during the study period according to the case identification algorithm. The case identification algorithm was guided by previously validated definitions used in epidemiological studies of IBD [16, 17]. IBD cases were considered confirmed if an individual had at least two IBD-related healthcare encounters on different days with an ICD-9 diagnosis code 555.x (CD) or 556.x (UC), or ICD-10 diagnosis code K50.x (CD) or K51.x (UC) [17]. Individuals with only one IBD diagnosis who also had at least one pharmacy claim for an IBD-specific medication (i.e., any aminosalicylic acid or biologic) (Table S1) on the day of or within ± 1 year from the IBD diagnosis code were also considered confirmed cases. This latter definition allowed us to capture persons with suspected IBD prior to the first recorded IBD diagnostic code. For persons with claims for both CD and UC, the IBD subtype was determined by the code used in the majority of claims over the most recent 1-year period. In cases lacking a majority, the most recent diagnosis was used to determine the subtype. All eligible patients were followed until the last date of enrollment in the health plan or study end (31 December 2019), whichever occurred first.

### Overall and study cohorts (2010–2019)

The overall General Population cohort included any eligible patient in the databases over the 10-year study period. Persons with missing identification data, year of birth, or sex information were excluded. The overall Prevalent UC cohort included any patient in the overall General Population cohort with UC confirmed during the study period using the case identification algorithm. The overall Incident UC cohort included any patient in the overall Prevalent UC cohort who had at least 12-month continuous enrollment prior to the index date during which no UC code, either diagnostic or pharmaceutical, was observed. In order to ensure the overall Incident UC cohort included only patients with their first qualifying UC code during their entire enrolment in the insurance plan, we applied an additional criterion of no prior UC code from the patients’ first observation in the databases, starting from year 2000 for CCAE and from year 2005 for JMDC, up until the index date.

The three overall cohorts were stratified into 10 sub-cohorts by calendar year. The calendar-year General Population cohort included any patient in the overall General Population cohort who was observed in the database during the corresponding calendar year. The calendar-year Prevalent UC cohort included any patient in the overall Prevalent UC cohort with one or more qualifying UC codes in the corresponding calendar year, irrespective of preceding years back to January 1, 2010. The calendar-year Incident UC cohort included any patient in the overall Incident UC cohort whose first qualifying UC code was observed in the database during the corresponding calendar year. Patients in the calendar-year cohorts served as denominators and numerators of annual prevalence/incidence estimations for the corresponding calendar year as appropriate.

### Variables

The index date for the overall Prevalent and Incident UC cohorts was the date of the first qualifying UC code, either diagnostic or pharmaceutical, observed during the 10-year study period.

The index date for the calendar-year Prevalent and Incident cohorts was the date of the first qualifying UC code observed in the corresponding calendar year.

Age and sex of patients in the overall Prevalent and Incident UC cohorts were determined on the respective index dates. Year of the first qualifying diagnosis and year of the first qualifying medication for UC in the overall Prevalent and Incident cohorts were extracted from the databases.

### Statistical analyses

Patient characteristics at cohort entry were presented descriptively using mean, standard deviation (SD), median, interquartile rage (IQR: Q1–Q3), and minimum and maximum values for continuous variables. Frequencies and percentages were reported for categorical variables which included age (< 6, 6– < 18, 18– < 45, and 45– < 65 years), sex (male, female), year of the first qualifying diagnosis, and year of the first qualifying medication. Differences between groups within each database were evaluated using *t*-tests for continuous variables and chi-square tests for categorical variables, with a *p*-value of < 0.05 as statistically significant.

Period prevalence per 100,000 population was estimated annually in each database using the calendar-year Prevalent UC cohort. For each calendar year, the numerator was the total number of unique patients in the calendar-year Prevalent UC cohort and the denominator was the total number of unique patients in the calendar-year General Population cohort. The denominators to estimate sex-specific and age group–specific prevalence were the total number of persons in the calendar-year General Population cohort in each sex strata and age group strata as appropriate.

Period incidence rates per 100,000 person-years were estimated annually in each database using the calendar-year Incident UC cohort. For each calendar year, the numerator was the total number of unique patients in the calendar-year Incident UC cohort. The denominator was the total person-years of at-risk population in the corresponding year, which was calculated as the total person-years of the calendar-year General Population cohort minus the total person-years of patients with prevalent UC in the corresponding year. The denominator also included at-risk period (i.e., person-years) of patients with incident UC in the year, from January 1 up until the index date. In order for the denominator to include only the at-risk population and not those patients who subsequently had the first qualifying UC code during any study year, we removed the person-years of incident UC cases that occurred in the preceding years. For each calendar year, the denominators to estimate sex-specific and age group–specific incidence rates were the total person-years of the at-risk population in each sex strata and age group strata in the corresponding year as appropriate.

Crude period prevalence and incidence rates estimated in each calendar year in the JMDC were directly standardized using the age distribution of the CCAE, which was used as the standard population for the corresponding year. Annual period prevalence (APP) and annual incidence rates (AIRs) across the 10-year study period were assessed for temporal trends using the Kendall Tau-*b* correlation, and the average of 10-year APPs and AIRs were estimated, respectively. The ratio and its 95% confidence interval (CI) of the average APP and AIR over the 10-year period were derived to compare the prevalence and incidence of UC in Japan (JMDC) to that in the US (CCAE).

The results of APP and AIR presented in the following section refer to crude estimates for the CCAE and age-standardized estimates for the JMDC unless otherwise stated. All analyses were performed using Aginity Workbench for Amazon Redshift (Version 4.9.3.2778) and SAS Enterprise Guide 7.15.

## Results

### Study population

**Fig. 1:**
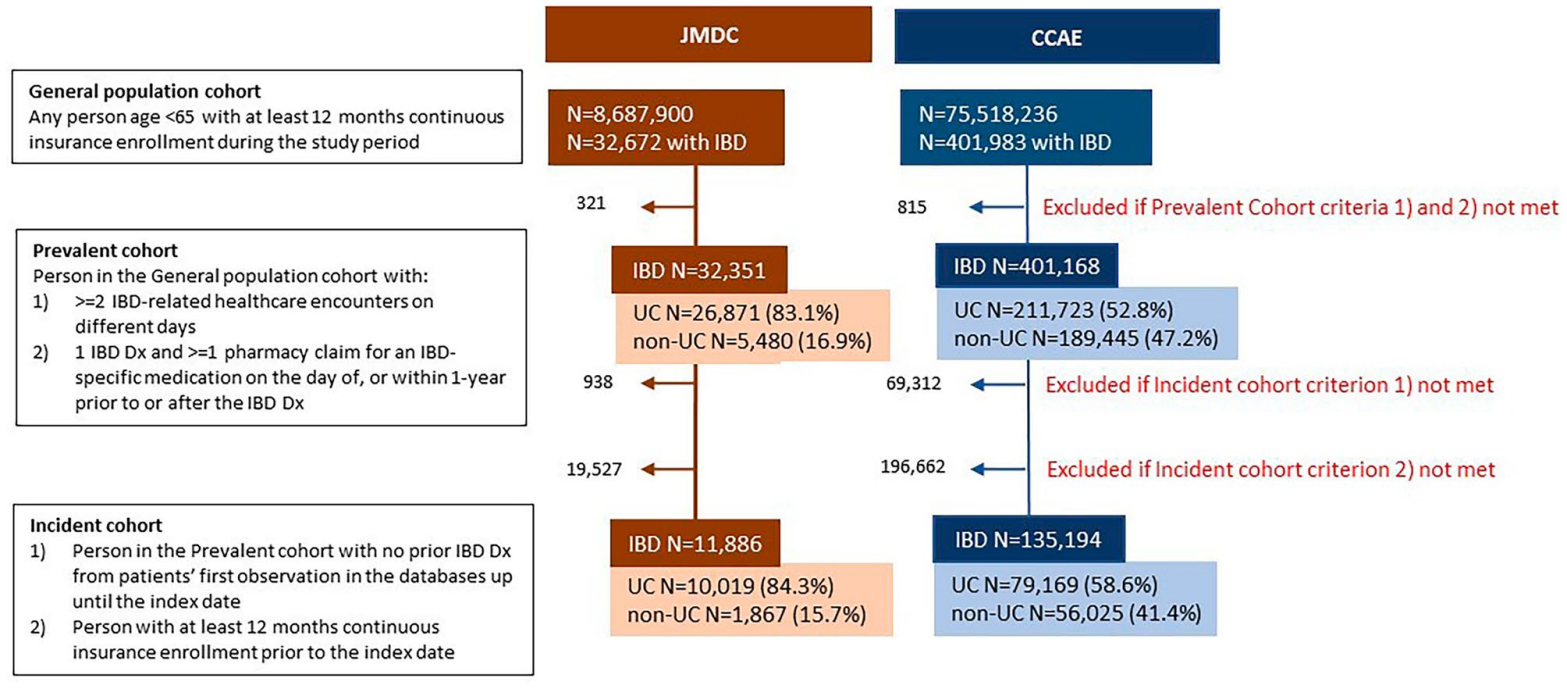
Patient entry into study cohorts in the JMDC (Japan) and CCAE (US) databases (January 1, 2010, to December 31, 2019). Abbreviations: CCAE, the IBM MarketScan Commercial Claims and Encounters; Dx, diagnosis; IBD, inflammatory bowel disease; JMDC, Japan Medical Data Center; N, number of patients; UC, ulcerative colitis

There were 32,351 unique prevalent IBD cases identified in the JMDC and 401,168 identified in the CCAE databases over the study period. Of these, 26,871 (81.3%) in JMDC and 211,723 (52.5%) in CCAE had a confirmed diagnosis of UC (Fig. 1). The Incident UC cohort comprised 10,019 patients in JMDC and 79,169 in CCAE with unique incident UC (Fig. 1). The majority of patients with newly diagnosed IBD in both databases had UC.

The mean age of patients was similar across the overall Prevalent and Incident cohorts in both databases (range 39.4 to 43.4 years), although the median age was lower in the JMDC (42 and 40.2 years in the overall Prevalent and Incident cohorts, respectively) than in the CCAE (45 and 46 years, respectively) (Table 1). Approximately 60.0% of patients in the overall Prevalent cohort and 63.6% in the overall Incident cohort in the JMDC were < 45 years of age at the index date, versus approximately 49.4% and 47.4%, respectively, in the CCAE. Men outnumbered women in both cohorts in the JMDC (61.0% and 63.8% in the overall Prevalent and Incident cohorts, respectively) whereas the reverse was observed in the CCAE (47.2% and 46.0%, respectively).

**Table 1:** Characteristics of patients with ulcerative colitis at the index date in the overall Prevalent and Incident UC cohorts in the JMDC (Japan) and CCAE (US) databases (January 2010–December 2019)

	**CCAE**		**JMDC**	
**Characteristic**	**Prevalent cohort**	**Incident cohort**	**Prevalent cohort**	**Incident cohort**
	**(*N* = 211,723)**	**(*N* = 79,169)**	**(*N* = 26,871)**	**(*N* = 10,019)**
**Age, years**				
Mean (SD)	43.2 (13.7)	43.4 (14.2)	40.9 (12.2)	39.4 (12.8)
Median	45	46	42	40.2
Min–max	1–64	1–64	0.5–64	1–64
Q1–Q3	33–55	33–55	32–50.1	30.0–49.2
**Age group, *n* (%)**				
< 6	412 (0.2)	195 (0.2)	52 (0.2)	42 (0.4)
6– < 18	6877 (3.2)	3243 (4.1)	788 (2.9)	513 (5.1)
18– < 45	97,414 (46.0)	34,137 (43.1)	15,271 (56.9)	5815 (58.1)
45– < 65	107,020 (50.6)	41,594 (52.6)	10,760 (40.0)	3649 (36.4)
**Sex, *n* (%)**				
Female	111,779 (52.8)	42,789 (54.0)	10,488 (39.0)	3624 (36.2)
Male	99,944 (47.2)	36,380 (46.0)	16,383 (61.0)	6395 (63.8)
**Year of first qualifying diagnostic code, *n* (%)**				
2010	46,273 (21.9)	8474 (10.7)	1571 (5.8)	160 (1.6)
2011	29,296 (13.8)	9879 (12.5)	799 (3.0)	222 (2.2)
2012	23,962 (11.3)	10,792 (13.6)	1141 (4.3)	369 (3.7)
2013	21,964 (10.4)	8066 (10.2)	2506 (9.3)	519 (5.1)
2014	18,323 (8.7)	8874 (11.2)	1259 (4.7)	742 (7.3)
2015	14,929 (7.0)	7516 (9.5)	3849 (14.3)	779 (7.8)
2016	15,406 (7.3)	7344 (9.3)	4291 (16.0)	1119 (11.2)
2017	14,588 (6.9)	6189 (7.8)	4669 (17.4)	1672 (16.7)
2018	14,959 (7.1)	5962 (7.5)	4585 (17.0)	2065 (20.6)
2019	12,023 (5.7)	6073 (7.7)	2201 (8.2)	2372 (23.8)
Total	211,723 (100)	79,169 (100)	26,871 (100)	10,019 (100)
Year of first qualifying pharmacy code*, *n* (%)				
2010	20,214 (9.5)	7387 (9.3)	741 (2.8)	139 (1.4)
2011	21,173 (10.0)	7993 (10.1)	701 (2.6)	179 (1.8)
2012	19,367 (9.1)	8327 (10.5)	1015 (3.8)	308 (3.1)
2013	18,884 (8.9)	7132 (9)	2225 (8.3)	455 (4.6)
2014	15,513 (7.3)	7057 (8.9)	1123 (4.2)	580 (5.8)
2015	12,786 (6.0)	5930 (7.5)	3398 (12.6)	676 (6.8)
2016	13,527 (6.4)	6035 (7.6)	3841 (14.3)	953 (9.5)
2017	12,947 (6.1)	5283 (6.7)	4249 (15.8)	1412 (14.1)
2018	13,175 (6.2)	4965 (6.2)	4324 (16.1)	1728 (17.2)
2019	10,162 (4.8)	4440 (5.6)	2208 (8.2)	2024 (20.2)
Total	157,788 (74.5)	64,549 (81.5)	23,825 (88.7)	8454 (84.4)

*CCAE* The IBM MarketScan Commercial Claims and Encounters (*CCAE* currently known as Merative™ MarketScan^®^), *JMDC* The Japan Medical Data Center, *N,* *n* number of patients, *q1–q3* interquartile range, *SD* standard deviation

*Pharmacy code used for case identification included UC-specific medication (i.e., any aminosalicylic acid or biologic) (see Table S1)

In the JMDC Incident cohort, the number of new cases of UC increased monotonically and markedly over time from 2010 to 2019. By contrast, the number of new cases in the CCAE Incident cohort increased until a peak in 2012 and subsequently decreased over time with the lowest number of cases in 2018.

The General Population cohort in JMDC consistently increased in size annually from 2010 to 2019 (7.2-fold) with a steep jump numerically from 2014 to 2015, while in the CCAE it increased from 2010 to 2012 (1.2-fold) and decreased thereafter (0.58-fold) (Table 2).

**Table 2:** Number of individuals in the calendar-year General Population cohort in the JMDC (Japan) and CCAE (US) databases (January 2010–December 2019)

**Calendar year**	**CCAE**	**JMDC**
2010	29,313,587	995,350
2011	33,761,752	1,288,784
2012	35,500,196	1,707,196
2013	29,428,503	2,677,818
2014	28,007,711	2,864,939
2015	23,502,775	4,210,042
2016	23,501,771	5,642,044
2017	21,554,060	6,826,557
2018	21,486,123	7,719,937
2019	20,751,889	7,181,382

*CCAE* The IBM MarketScan Commercial Claims and Encounters (CCAE currently known as Merative™ MarketScan^®^), *JMDC* The Japan Medical Data Center, *N* number of patients

### Period prevalence of UC

Crude period prevalence rates of UC in the JMDC increased annually from 158 per 100,000 population in 2010 to 266 per 100,000 population in 2019.

After age standardization, the average of the APP of UC over the 10-year period was 56.0 (95% CI 5.0–98.0) per 100,000 population in the JMDC and 199.6 (95% CI 157.9–232.9) per 100,000 population in the CCAE. The ratio of average APPs for UC for JMDC over CCAE was 0.28 (95% CI 0.22–0.34), indicating that the APP was on average 72% lower in the JMDC compared to the CCAE (*p* < 0.0001) over the 10-year study period.

**Fig. 2:**
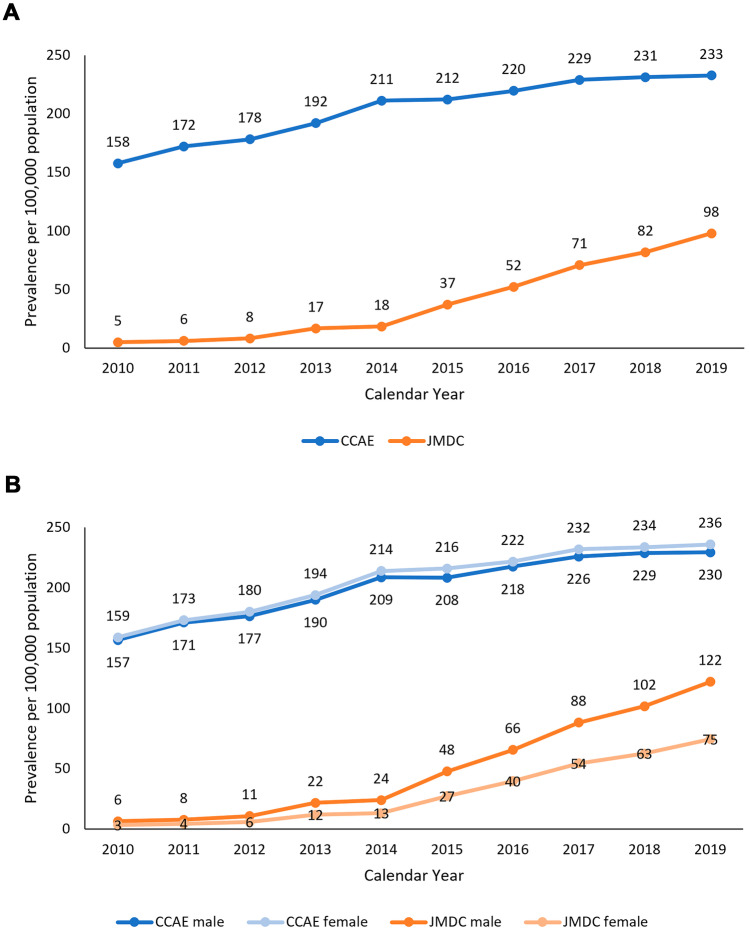
Annual period prevalence* (per 100,000 population) of UC in the JMDC (Japan) and CCAE (US) databases. **A** Overall. **B** By Sex. *Crude (unweighted) estimates in the CCAE and age-standardized estimates in the JMDC using direct standardization based on the CCAE’s age distribution

The prevalence rates of UC increased significantly over time in both databases (*p* < 0.0001 for CCAE, *p* < 0.0001 for JMDC) (Fig. 2A). There was no difference in trends between men and women in either database (JMDC *p* = 0.2280 and CCAE *p* = 0.7133) during the study period (Fig. 2B).

**Fig. 3:**
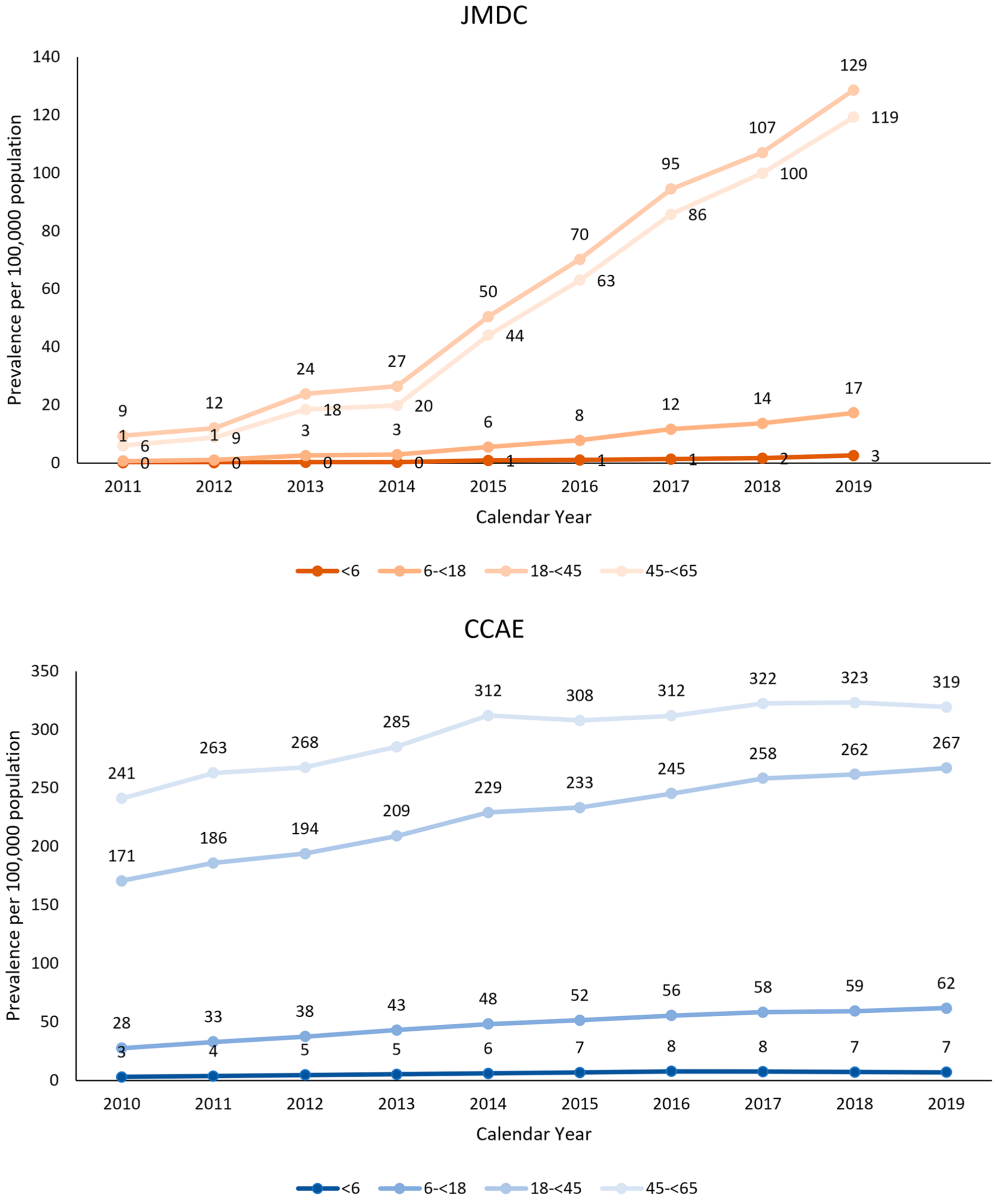
Annual period prevalence* (per 100,000 population) of UC in the JMDC (Japan) and CCAE (US) databases by age group. *Crude (unweighted) estimates in the CCAE and age-standardized estimates in the JMDC using direct standardization based on the CCAE’s age distribution

There were marked differences in the age distribution of prevalent UC cases in the two databases (Fig. 3). In the JMDC, the prevalence of UC increased significantly over time in each age group (*p* ≤ 0.0001 for all age groups) and the increases were significantly different across age groups (*p* ≤ 0.0001). However, the increase was highest in adult age groups, with a rapid increase from 2015. The highest prevalence was observed in 18– < 45 followed by 45– < 65 year-olds.

As observed in the JMDC, the prevalence of UC in the CCAE increased significantly over time in each age group (*p* ≤ 0.0024 for all age groups). However, no difference was observed across age groups (*p* = 0.3783). The prevalence of UC was highest in adult age groups, with the highest prevalence in the 45– < 65-year-old group.

The APP of UC in adults (≥ 18) increased more in women than in men in the JMDC (*p* = 0.0001), whereas the APP increased similarly among women and men in the CCAE (*p* = 0.8579) (Fig. S1).

Visual inspection of prevalence with more granular age stratification (5-year increments) showed a general shift in the age group with the highest prevalence of UC over time in the CCAE. The highest UC prevalence was in 45– < 50 and 60– < 65 year-olds in 2010, with an increase in prevalence in younger age groups so that age groups with the highest UC prevalence included 35– < 40 and 55– < 60 year-olds in both men and women in later calendar years. By 2017, there was little variation in prevalence across age groups covering 30–60 year-olds (Fig. S2-1).

The same trend was not observed in the JMDC. Age groups with the highest prevalence were 35– < 45 year-olds from 2010 through 2014. Thereafter, the highest prevalence was observed in 40– < 45 year-olds (Fig. S2-2).

### Incidence of UC

**Fig. 4:**
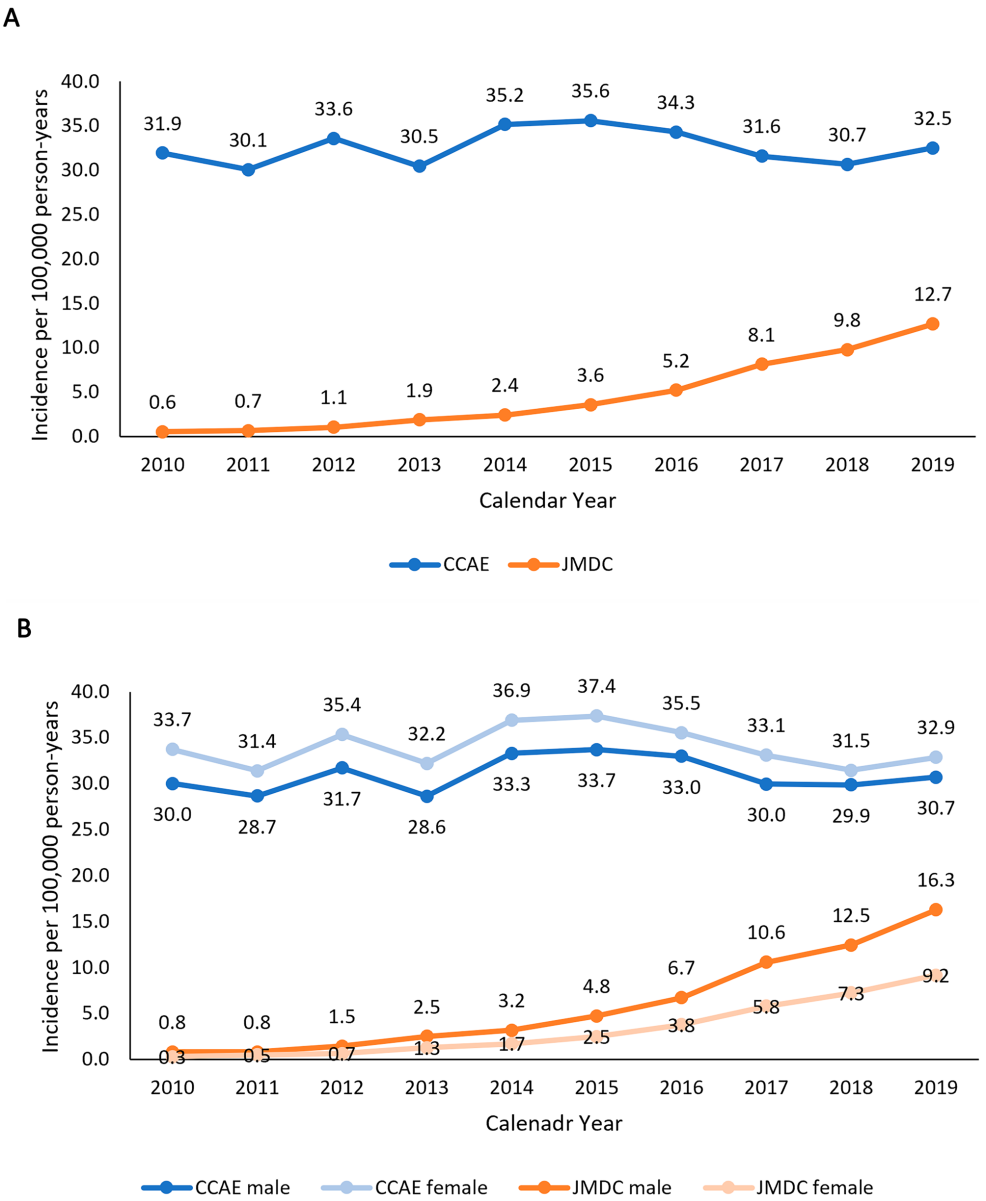
Annual incidence rates* (per 100,000 person-years) of UC in the JMDC (Japan) and CCAE (US) databases. **A** Overall. **B** By sex. *Crude (unweighted) rates in the CCAE and age-standardized rates in the JMDC using direct standardization based on the CCAE’s age distribution

The AIR of UC increased each year in the JMDC, from 0.6 per 100,000 person-years in 2010 to 12.7 per 100,000 person-years in 2019 (*p* < 0.0001). By contrast, there was no evidence for any temporal trends in the CCAE, with an AIR 31.9 per 100,000 person-years in 2010 and 32.5 per 100,000 person-years in 2019 (*p* = 0.6547) (Fig. 4A).

The average of the AIR of UC over the 10-year period was lower in the JMDC (6.6 per 100,000 person-years) than in the CCAE (32.5 per 100,000 person-years) (*p* < 0.0001). The ratio of the average AIRs was 0.20 (95% CI 0.06–0.34; *p* = 0.0020), indicating the incidence of UC was on average 80% lower in the JMDC than in the CCAE.

The AIRs of UC were higher in men in the JMDC and higher in women in the CCAE (Fig. 4B). In the JMDC, the AIR increased by 29-fold in women (*p* < 0.0001) over the study period, versus 19-fold (*p* < 0.0001) in men.

The highest AIRs for UC throughout the study period were observed in the CCAE in women (average AIR = 34.0, range: 31.4–37.4), followed by men (average AIR = 30.9, range: 28.6–33.7). In the JMDC, AIRs were higher in men (average AIR = 8.5, range: 0.8–16.3) than in women (average AIR = 4.7, range: 0.3–9.2). The ratio of average AIRs for UC was 0.19 (95% CI 0.06–0.32) for men and 0.10 (95% CI 0.03–0.17) for women, respectively, indicating the AIRs for UC among Japanese men and women were respectively 81% and 90% lower than those of their counterparts in the US.

**Fig. 5:**
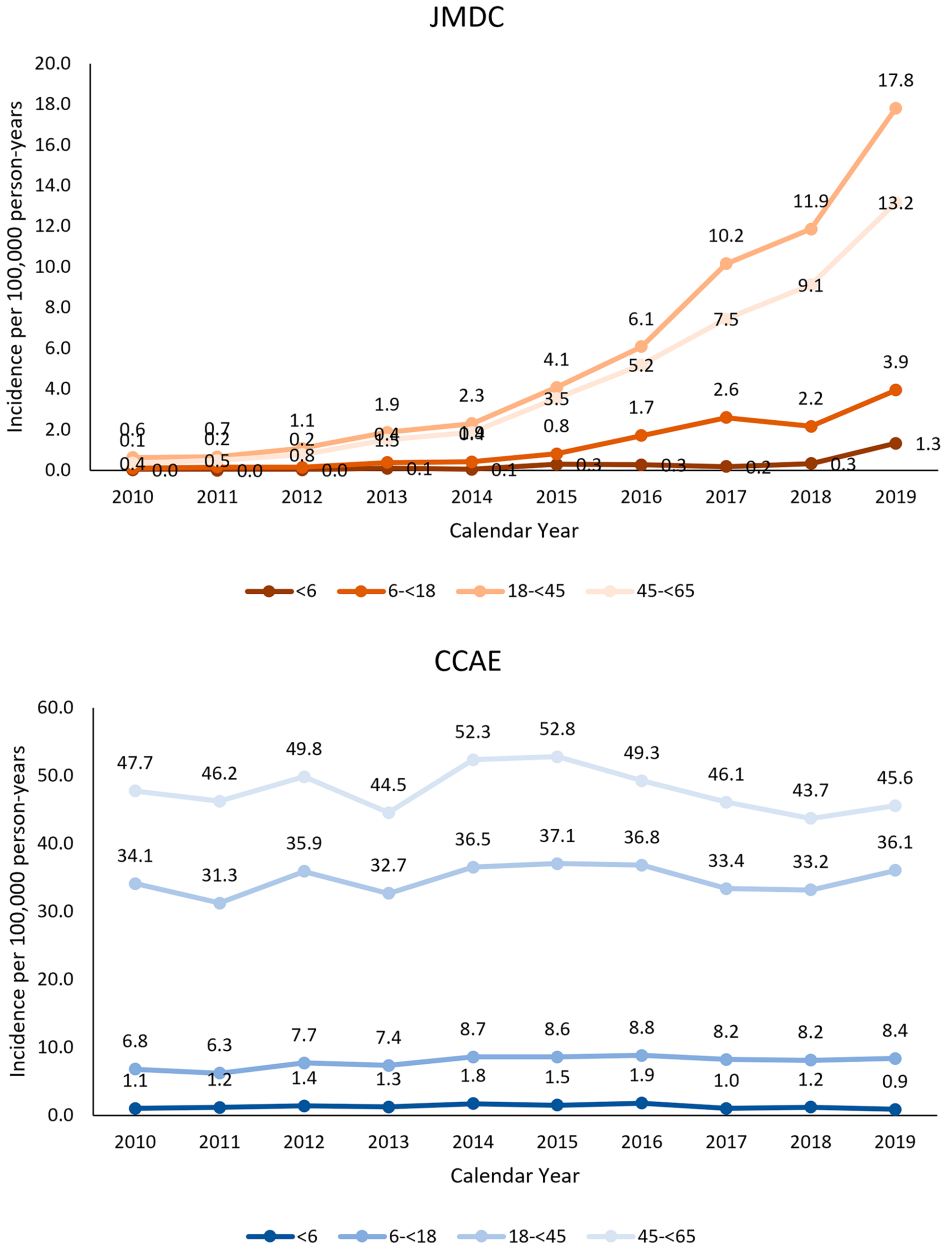
Annual incidence rates* (per 100,000 person-years) of UC in the JMDC (Japan) and CCAE (US) databases by age group. *Crude (unweighted) rates in the CCAE and age-standardized rates in the JMDC using direct standardization based on the CCAE’s age distribution

Statistically significant increases in the incidence of UC were observed for all age groups in the JMDC (*p* ≤ 0.017 for all age groups) and the increases were significantly different among 4 age groups (*p* < 0.0001) (Fig. 5). However, the relative increase was highest in the 45– < 65 year age group (37-fold increase between 2010 and 2019). Throughout the study period, the annual incidence of UC was highest in the 18– < 45 year age group (average AIR = 7.9, range: 0.6–17.8) and lowest in children < 6 years of age (AIR = 0.4, range: 0.03–1.33).

Consistent with the apparent stability of the incidence of UC in the CCAE over the study period, there was no evidence for any temporal trends in any age group (*p* ≥ 0.3252) except for the 6– < 18 year age group (*p* = 0.0141) for which the incidence rate increased modestly by 1.3-fold (Fig. 5). Overall, the rates in patients 18 years and older were higher than those in younger patients; however, there was no difference in the increases over time across all age groups (*p* = 0.9725).

Age-specific AIRs stratified by age group and gender reflected the findings above (Fig. S3).

## Discussion 

We analyzed temporal trends in the prevalence and incidence of UC in two diverse developed countries, one Western and one Asian, using two employment-based claims databases that covered similar populations—employees of large corporations and their dependents.

There were marked differences in the epidemiology of UC between the two countries: patients with UC in Japan tended to be younger than those in the US, and men were affected more than women in Japan, whereas the reverse was true in the US. We observed a marked and consistent increase in the prevalence and incidence of UC in Japan that is in line with previous reports [10, 18].

Crude UC prevalence in Japan was 211 per 100,000 population in 2014 which is in line with previous crude estimates of 133.2–172.9 per 100,000 population in 2014 obtained through national surveys [10, 18]. By contrast, the age-standardized prevalence was 18 per 100,000 population in 2014 reflecting direct standardization using the CCAE age distribution to specifically allow comparison of the two populations. The rate of change in age-standardized prevalence and incidence in Japan appeared to increase substantially from 2015. This may be due to real changes in prevalence and incidence of UC before and after 2015; however, we cannot exclude that awareness, disease diagnosis, and ascertainment of IBD subtypes among gastroenterologists increased after publication of the Second Edition of the Evidence-based Clinical Practice Guidelines for Inflammatory Bowel Disease in 2016 [19], which included marked changes compared to the First Edition (2006). We can also not exclude an impact due to the different trends in the size of the General Population cohort in the JMDC, reflecting the trends of the total enrollment and coverage of the JMDC, relative to that of the CCAE in the same period (Table 2), which may in turn have had influences in weights for direct standardization which were noticeably different in the pre-2015 period compared to 2015 and after.

Incidence rates climbed annually in Japan, with relatively higher increases in women than in men and in the 6– < 18 year age group, which is in line with previously reported trends in Japan and elsewhere [10, 20]. Of note, the population > 15 years of age are treated as adults in clinical practice in Japan, and more approved medications for UC indication became available in the later calendar years of the study, including new drugs, supplemental approvals for new formulations, and expansion of indications of some drugs to include the pediatric population from 6 years of age (Table S1). These expanded treatment options might have influenced physician’s clinical decision-making and diagnosis attribution overall in the 6– < 18 year age group. The availability of a larger number of pharmacy codes may also have increased the ability of our algorithm to capture cases of UC, leading to increased incident cases in later study years.

A systematic review of 131 population-based studies from 48 countries (2010–February 2020) reported increasing incidence of pediatric-onset IBD (age < 21) [20], and rates of very early onset (< 6 years of age) UC that were highest in North America, followed by Europe, and were lowest in Asia for which our findings comparing the US and Japan support this observation.

Prevalence and incidence rates of UC were substantially higher in the US than in Japan in all study years, in all age groups, and in both sexes. The prevalence of UC increased annually in the US, consistent with a previous report using two US healthcare claims databases between 2007 and 2016 overlapping with our study period [8]. In that study, the pooled prevalence of UC in the US was 181.1 per 100,000 population in 2016, which is similar to our estimate of 220 per 100,000 in the same year. Prevalence increased similarly in men and women, and in the age groups between 6 and < 65 years of age. In contrast to the strong temporal trends evident in Japan, the incidence of UC in the US remained stable over the study period, with no evidence of changes over time in men versus women, or in any age group except for a modest increase in 6– < 18 year-olds. Increasing treatment options for UC during the study period occurred in the US as well, including orphan drug designations for pediatric populations, which might have influenced diagnostic trends observed in children, as well as UC case detection overall in this study (Table S1). Results from the 6– < 18 years old age group are consistent with an earlier report of a 2.7-fold increase in the incidence of UC in US children < 18 years of age between 1996 and 2006 [21]. Our results contrast with a population-based study conducted in Olmsted County in the US between 1970 and 2010, which showed a higher incidence rate of UC in men than in women and in the 20–29 year age group [7]. It is not clear if this represents a change in the epidemiology of UC in the US before and after 2010, or underlying differences in the study populations due to the different study designs and data sources.

Kaplan and Windsor [22] recently proposed that the global epidemiology of IBD could be classified into four epidemiological stages: emergence, acceleration in incidence, compounding prevalence, and prevalence equilibrium. The authors classified newly industrialized countries in Asia as established in the second stage of IBD evolution, which is characterized by rapidly increasing incidence while prevalence remains low, as we observed in Japan. The authors put forward two main factors driving the second stage: a diagnostic bias reflecting increased awareness, improved diagnosis, improved access to health care, and improved disease surveillance; and a true rise in incidence where individuals with mutated genetic susceptibility are exposed to environmental factors resulting from Westernization and potential impacts on the gastrointestinal microbiome [22]. The former is evident in Japan as shown by periodic updates in Evidence-based Clinical Practice Guidelines for IBD conducted every 2–3 years since 2016 [19, 23, 24], updated pediatric guidelines published in 2019 [25], increasing research focused on IBD that recently received funding from the Ministry of Health, Labour, and Welfare, and the establishment of IBD registries [26].

Kaplan and Windsor predicted that over the next three decades, the increase in incidence followed by the exponential rise in prevalence in newly industrialized countries in Asia will echo the same transition observed in Western countries over the latter half of the twentieth century, which was followed by stabilizing incidence and compounding prevalence. Our findings for the US are consistent with a “compounding prevalence” stage characterized by onset at a young age with a subsequent, steady increase in population prevalence.

Strengths of our study include the use of two healthcare databases that cover similar population in two diverse countries with advanced healthcare systems, the large sample size, granular age stratification including under 6 year-olds, strict incident UC identification capturing only cases never diagnosed back to the earliest records in the databases, and the 10-year observation period which allowed detection of temporal trends in both countries. Furthermore, direct standardization allowed comparison of the two populations. Potential limitations are inherent differences between the database populations due to possible selection bias given that they only include insured employees and their dependents. Older persons and individuals with severe UC may be unable to work and are less likely to be captured in employment-based claims databases. It is therefore possible that the demographic characteristics of patients in both countries were biased toward healthier population not representative of the total population of patients with UC in each country. Healthcare-seeking and medication-use behaviors may differ in differently insured, underinsured, or uninsured persons. The study design may have led to the prevalence for 2010 and 2019 to be underestimated. Patients in these years were not included when potentially qualifying UC codes to establish confirmed UC were outside the study period (i.e., codes in 2009 or 2020). We excluded the population aged over 65 years and additional analyses using different data sources may provide further insights on these older and high-risk populations. The absence of clinical details in claims databases increases the risk of diagnostic misclassification. We minimized this risk by identifying cases using a combination of diagnostic codes and UC-specific mediation codes. Another limitation is the absence of information on race/ethnicity, socioeconomic status, and geographic location of the study population in the respective databases, which may have had more impact on the results in the US because of the more diverse population in that country.

In conclusion, individuals with UC in Japan and the US differ in their demographic characteristics, suggesting differences in genetic or environmental factors that alter the risk of disease. The epidemiology of UC is evolving rapidly in Japan, with incidence and prevalence that are increasing markedly. While the incidence rate of UC appears to have stabilized in most age groups in the US, prevalence and the incidence in children and adolescents continue to increase. The observed increase in incidence among pediatric populations in both countries is of particular concern. The data point to a growing disease burden in both countries that warrants urgent attention in terms of prevention and management.

### Supplementary Information

Below is the link to the electronic supplementary material.

Supplementary file1 (DOCX 234 KB)

## Data Availability

The data underlying this article were provided by the JMDC and CCAE under license. Data will be shared on request to the corresponding author with permission of JMDC and/or CCAE.
