# Supplementary material for: Trends in the prevalence and incidence of ulcerative colitis in Japan and the US
Source: Int J Colorectal Dis. 2023 May 19;38(1):135. doi: 10.1007/s00384-023-04417-6 (PMC10198866; doi:10.1007/s00384-023-04417-6)
Supplement: Supplementary file 1 — Supplementary file1 (DOCX 234 KB) [file 384_2023_4417_MOESM1_ESM.docx]

# **Trends in the Prevalence and Incidence of Ulcerative Colitis in Japan and the United States**

Michiyo Yamazaki, Hsingwen Chung, Youran Xu, Hong Qiu

**Supplementary material**

**Fig. S1** Period Prevalence* (per 100,000 population) of UC stratified by sex and age in the JMDC (Japan) and CCAE (US) databases


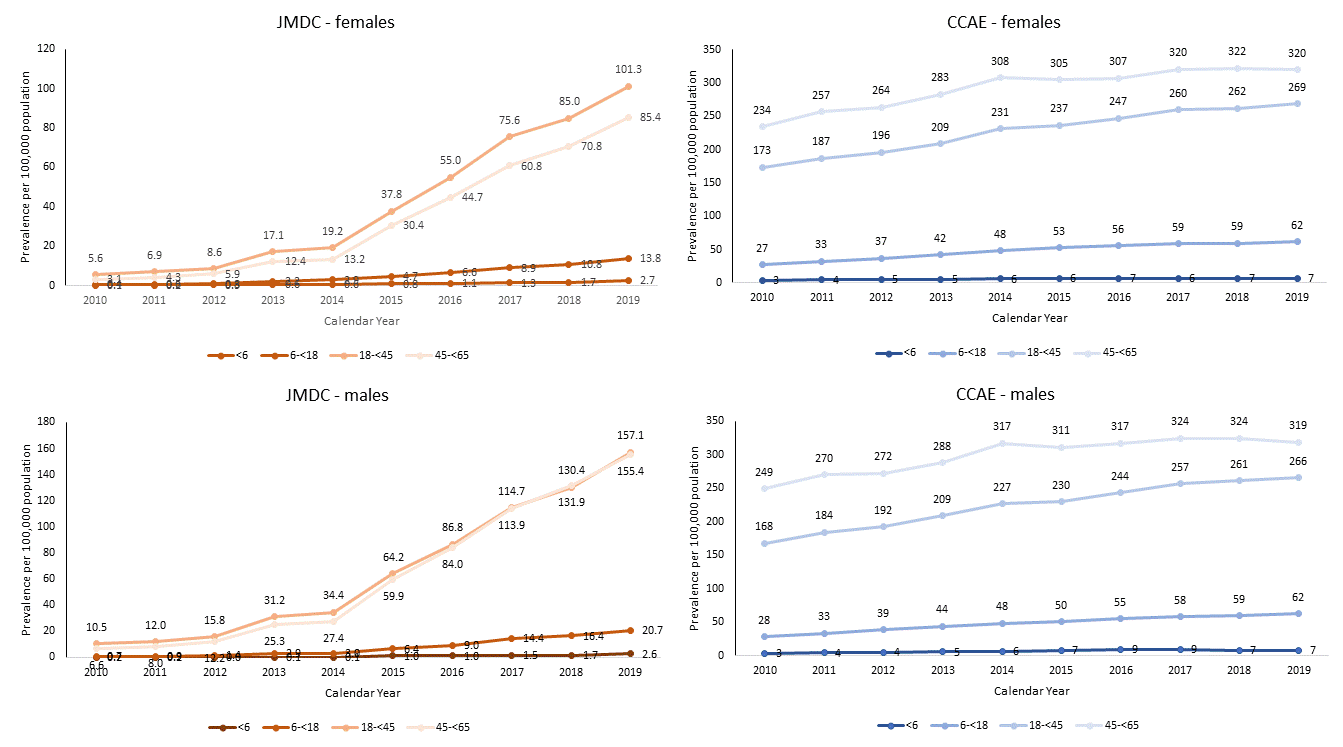


*crude (unweighted) rates in the CCAE and age-standardized rates in the JMDC using direct standardization based on the CCAE’s age distribution.

**Fig. S2-1** Annual Period Prevalence per 100,000 population in the CCAE (US)


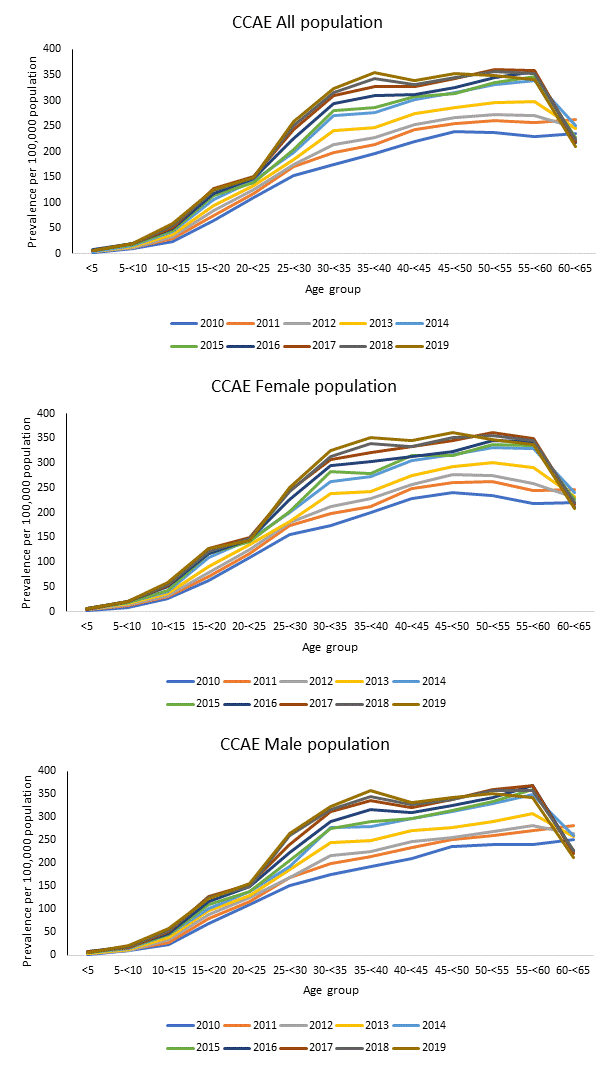


**Fig. S2-2** Annual Period Prevalence* per 100,000 population in the JMDC (Japan)


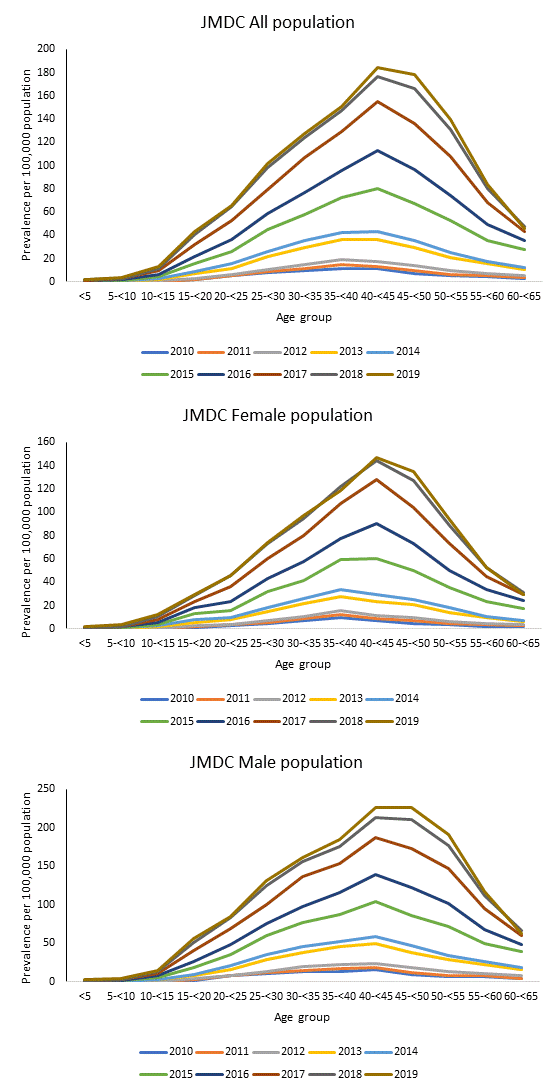


*age-standardized rates in the JMDC using direct standardization based on the CCAE’s age distribution.

**Fig S3** Age-specific Annual Incidence Rates* (per 100,000 person-years) of UC in the JMDC (Japan) and CCAE (US) databases stratified by sex


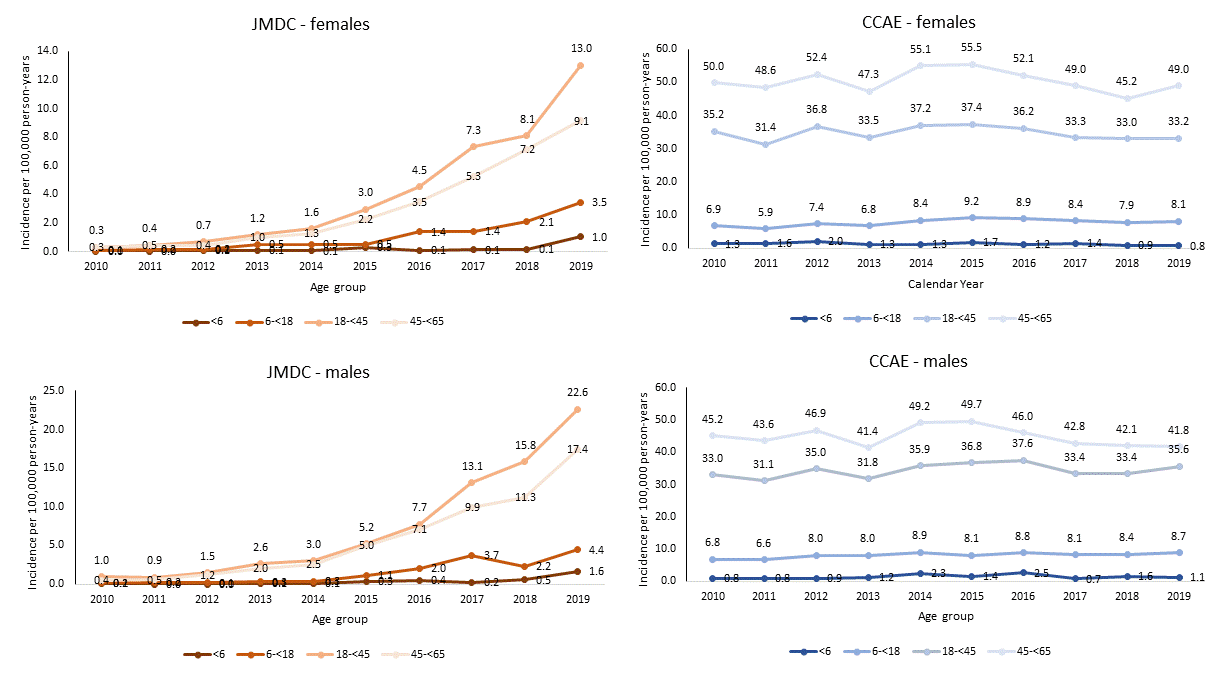


*crude (unweighted) rates in the CCAE and age-standardized rates in the JMDC using direct standardization based on the CCAE’s age distribution.

**Table S1** UC-specific Medications Available During the Study Period (2010-219) in the US and Japan and the Date of Approval

| **Drug category** | **ATC code** | **Drug (generic name)** | **Year of first approval for UC indication** | |
| --- | --- | --- | --- | --- |
|  |  |  | **The US** | **Japan** |
| 5-ASA | A07EC01 | Sulfasalazine | 1950^[[1]](#endnote-1)^ | 1969^[[2]](#endnote-2)^ |
|  | A07EC02 | Mesalazine | 1987^[[3]](#endnote-3)^ | 1996^[[4]](#endnote-4)^ |
|  | A07EC03 | Olsalazine | 1990^[[5]](#endnote-5)^ | - |
|  | A07EC04 | Balsalazide | 2000^[[6]](#endnote-6)^ ^[[7]](#endnote-7)^ ^a^ | - |
| Biologic | L04AB02 | Infliximab | Sep 2005^[[8]](#endnote-8)^ | Jun 2010^[[9]](#endnote-9)^ ^[[10]](#endnote-10)^ ^b^ |
|  | L04AB04 | Adalimumab | Sep 2012^[[11]](#endnote-11)^ ^c^ | Jun 2013^[[12]](#endnote-12)^ |
|  | L04AB06 | Golimumab | May 2013^[[13]](#endnote-13)^  ^c^ | Mar 2017^[[14]](#endnote-14)^ |
|  | L04AA33 | Vedolizumab | May 2014^[[15]](#endnote-15)^ | Jul 2018^[[16]](#endnote-16)^ |
|  | L04AC05 | Ustekinumab | Oct 2019^[[17]](#endnote-17)^  ^c^ | -  ^d^ |

Abbreviations: 5-ASA= ATC=The Anatomical Therapeutic Chemical code; UC= ulcerative colitis; US=United States.

Footnotes:
a: indication expansion for 5 years of age and older in 2006; b: infliximab was approved for 6 years of age and older in 2017; c: orphan designation for pediatric population during the study period (adalimumab was approved for pediatric population 5-<17 years of age in 2021, outside the study period); d: ustekinumab was approved in 2020 (outside the study period).

1. https://www.accessdata.fda.gov/scripts/cder/daf/index.cfm?event=overview.process&ApplNo=007073 [↑](#endnote-ref-1)
2. Real life results in using 5-ASA for maintaining mild to moderate UC patients in Japan, a multi-center study, OPTIMUM Study. Nagahori et al. BMC Gastroenterology (2017) 17:47. DOI 10.1186/s12876-017-0604-y [↑](#endnote-ref-2)
3. https://www.accessdata.fda.gov/scripts/cder/daf/index.cfm?event=overview.process&ApplNo=019618 [↑](#endnote-ref-3)
4. Real life results in using 5-ASA for maintaining mild to moderate UC patients in Japan, a multi-center study, OPTIMUM Study. Nagahori et al. BMC Gastroenterology (2017) 17:47. DOI 10.1186/s12876-017-0604-y [↑](#endnote-ref-4)
5. <https://www.accessdata.fda.gov/scripts/cder/daf/index.cfm?event=BasicSearch.process> [↑](#endnote-ref-5)
6. https://www.accessdata.fda.gov/scripts/cder/daf/index.cfm?event=overview.process&ApplNo=020610 [↑](#endnote-ref-6)
7. https://www.accessdata.fda.gov/drugsatfda_docs/appletter/2006/020610s016ltr.pdf [↑](#endnote-ref-7)
8. https://www.accessdata.fda.gov/drugsatfda_docs/appletter/2005/103772_5113ltr.pdf [↑](#endnote-ref-8)
9. https://www.pmda.go.jp/files/000232774.pdf [↑](#endnote-ref-9)
10. https://www.mt-pharma.co.jp/news/assets/pdf/MTPC170518.pdf [↑](#endnote-ref-10)
11. https://www.accessdata.fda.gov/drugsatfda_docs/appletter/2012/125057Orig1s232ltr.pdf [↑](#endnote-ref-11)
12. https://www.pmda.go.jp/files/000232771.pdf [↑](#endnote-ref-12)
13. https://www.accessdata.fda.gov/drugsatfda_docs/appletter/2013/125289Orig1s077,s078,s079ltr.pdf [↑](#endnote-ref-13)
14. https://www.pmda.go.jp/files/000232770.pdf [↑](#endnote-ref-14)
15. https://www.accessdata.fda.gov/drugsatfda_docs/appletter/2014/125476Orig1s000ltr.pdf [↑](#endnote-ref-15)
16. https://www.pmda.go.jp/files/000235288.pdf [↑](#endnote-ref-16)
17. https://www.accessdata.fda.gov/drugsatfda_docs/appletter/2019/761044Orig1s003,%20125261Orig1s152ltr.pd [↑](#endnote-ref-17)
